# Causal Effect of Dietary Patterns on Cerebral Small Vessel Disease: A Mendelian Randomization Study

**DOI:** 10.1002/fsn3.71374

**Published:** 2026-02-04

**Authors:** Yong Zeng, Ziqian Zhao, Hongyan Liu, Yijun Zeng, Song Xu, Shanjing Nie

**Affiliations:** ^1^ Department of Neurosurgery The Third Affiliated Hospital of Chengdu Medical College, Chengdu Pidu District People's Hospital Chengdu Sichuan China; ^2^ Institute of Brain Science and Brain‐inspired Research Shandong First Medical University & Shandong Academy of Medical Sciences Jinan Shandong China; ^3^ Department of Neurology The Third Affiliated Hospital of Chengdu Medical College, Chengdu Pidu District People's Hospital Chengdu Sichuan China; ^4^ Department of Geriatric Neurology, Anti‐Aging Monitoring Laboratory Shandong Provincial Hospital Affiliated to Shandong First Medical University Jinan Shandong China

**Keywords:** causal association, cerebral small vessel disease, dietary intake, Mendelian randomization

## Abstract

While emerging observational evidence suggests associations between dietary factors and cerebral small vessel disease (CSVD), the causal nature of these relationships remains unestablished. This study employed a two‐sample Mendelian randomization (MR) framework to investigate genetically predicted causal effects of dietary patterns on neuroimaging markers of CSVD. We utilized Genome‐Wide Association Study (GWAS) summary statistics from European‐ancestry cohorts for 32 dietary exposures and four CSVD phenotypes: white matter hyperintensities (WMH), lacunar strokes (LS), enlarged perivascular spaces (PVS), and brain microbleeds (BMB). Specifically, WMH volume, fractional anisotropy (FA), and mean diffusivity (MD) are indicators related to WMH. Genetic instruments (single‐nucleotide polymorphisms, SNPs) were rigorously selected using genome‐wide significance thresholds (*p* < 5 × 10^−8^/*p* < 5 × 10^−6^), linkage disequilibrium clumping (*r* < 0.001), and pleiotropy exclusion criteria. The primary analysis employed inverse‐variance weighted (IVW) MR, with validation through four complementary methods: weighted median, MR‐Egger regression, weighted mode, and simple mode. Significant associations (*p* < 0.05) underwent false discovery rate (FDR) correction. Robustness was further assessed through heterogeneity testing (Cochran's Q), horizontal pleiotropy evaluation (MR‐Egger intercept), and leave‐one‐out analysis. Simultaneously perform reverse MR analysis to eliminate reverse causal relationships. Our MR analysis revealed several genetically predicted causal associations between dietary factors and CSVD neuroimaging markers. Higher polyunsaturated fatty acid (PUFA) levels demonstrated a protective effect against WMH volume (β = −0.070, 95% CI: −0.126 to −0.015; se = 0.028; *p* = 0.013, FDR‐adjusted *p* = 0.033). Similarly, our analysis revealed a protective causal association between monounsaturated fatty acid (MUFA) intake and the risk of lobar BMB (β = −0.298, se = 0.115, 95% CI: −0.523 to −0.073; *p* = 0.009, FDR‐adjusted *p* = 0.035). Conversely, increased iron intake exhibited detrimental effects on both any BMB (β = 0.247, se = 0.094, 95% CI: 0.064 to 0.430; *p* = 0.008, FDR‐adjusted *p* = 0.041) and strictly deep BMB (β = 0.414, se = 0.153, 95% CI: 0.116 to 0.713; *p* = 0.007, FDR‐adjusted *p* = 0.033). Notably, suggestive protective associations of coffee consumption (β = −0.088, se = 0.044, 95% CI: −0.173 to −0.002; *p* = 0.045, FDR‐adjusted *p* = 0.225) and non‐oily fish intake (β = −0.193, se = 0.098, 95% CI: −0.386 to −0.001; *p* = 0.049, FDR‐adjusted *p* = 0.248) with basal ganglia PVS were observed; however, these associations did not survive multiple testing correction (FDR‐adjusted *p* > 0.05). All significant findings showed no evidence of heterogeneity (*PS*__heterogeneity_ > 0.05) or horizontal pleiotropy (*PS*__pleiotropy_ > 0.05). Reverse MR analyses revealed no causal effects of CSVD features on dietary exposures (*PS* > 0.05). This study provides novel genetic evidence supporting heterogeneous causal effects of dietary patterns on distinct CSVD phenotypes. The protective effect of PUFA against white matter injury, together with the beneficial role of MUFA in brain microbleeds, contrasted with iron's detrimental impact on brain microbleeds, underscoring the pathophysiological complexity of diet‐CSVD interactions. While nominally significant associations between coffee/fish intake and PVS require further validation, our findings emphasize the potential of targeted nutritional interventions in CSVD prevention. Future prospective studies with standardized dietary assessments and longitudinal neuroimaging are warranted to translate these genetic insights into clinical practice.

## Graphical abstract

This Mendelian randomization (MR) study reveals causal links between dietary factors and cerebral small vessel disease (CSVD) neuroimaging markers. Genetically predicted higher polyunsaturated fatty acid (PUFA) levels reduce white matter hyperintensity volume, while monounsaturated fatty acid (MUFA) intake lowers lobar brain microbleeds (BMB) risk. In contrast, increased iron intake elevates BMB risk. These findings support nutrient‐specific dietary interventions for CSVD prevention.

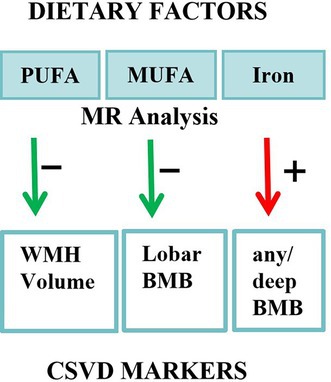

## 1 Introduction

Cerebral small vessel disease (CSVD) is a neurovascular disorder characterized by structural and functional abnormalities in cerebral microarteries, capillaries, and venules. As the leading vascular risk factor for ischemic stroke and vascular dementia, CSVD also contributes significantly to gait disturbances, postural instability, and affective disorders in the elderly population (Wardlaw et al. 2013). Its clinical manifestations exhibit marked heterogeneity, ranging from subclinical neuroimaging biomarkers to disabling strokes and vascular cognitive impairment (Pasi and Cordonnier 2020). Pathologically, CSVD involves blood–brain barrier (BBB) dysfunction, endothelial impairment, and neuroinflammatory cascades (Walker et al. 2017). Current diagnosis relies on magnetic resonance imaging (MRI) biomarkers, including recent small subcortical infarcts, lacunar stroke (LS), white matter hyperintensities (WMH), brain microbleeds (BMB), enlarged perivascular spaces (PVS), and cerebral atrophy (Wardlaw et al. 2013). Advanced techniques like diffusion tensor imaging (DTI) further enable microstructural characterization through parameters such as fractional anisotropy (FA) and mean diffusivity (MD), revealing early CSVD‐related tissue alterations (Pasi et al. 2016). Despite its high prevalence and potential therapeutic window, targeted interventions grounded in pathophysiology remain elusive. Epidemiological studies have established robust associations between dietary patterns and chronic diseases, including cerebrovascular disorders (Niu et al. 2024; Zhao et al. 2023). Unhealthy dietary patterns (e.g., high salt and ultra‐processed foods) exacerbate vascular injury via oxidative stress and pro‐inflammatory gene activation, while deficiencies in essential fatty acids, antioxidant vitamins, and other elements are closely associated with systemic inflammation‐oxidative (Furman et al. 2019). Dietary patterns are a crucial reserve variable for preventing CSVD (Liu et al. 2023; Gardener et al. 2012; Song et al. 2022), but there is a lack of evidence from large randomized trials (Wardlaw et al. 2019, 2021). Observational studies have pointed to associations between dietary patterns and the characteristics of CSVD, suggesting that healthy eating habits may slow the progression of CSVD. These findings suggest that dietary interventions could be a new strategy for the prevention and treatment of CSVD. However, the effectiveness of causal inference is limited by the inherent biases in the observational study design, including residual confounding factors and reverse causality. Mendelian randomization (MR) analysis addresses these constraints by leveraging genetic variants as instrumental variables to emulate randomized controlled trials. This approach capitalizes on the random assortment of alleles during meiosis, effectively minimizing confounding biases and reverse causation inherent in traditional observational studies.

MR employs single nucleotide polymorphisms (SNPs) as genetic instrumental variables (IVs) to infer causal relationships between exposures and outcomes. This approach capitalizes on the random transmission of alleles during gamete formation, ensuring that genetic variants are distributed independently of environmental confounders (Davies et al. 2018). In two‐sample MR designs, exposure and outcome associated genetic effects are derived from separate Genome‐Wide Association Study (GWAS) summary statistics (Lawlor 2016). Recent advancements in GWAS, which particularly the availability of large‐scale neuroimaging‐derived CSVD phenotypes from biobanks (e.g., WMH volume quantification, BMB), now enable MR analyses with sufficient statistical power to detect modest but clinically meaningful effects. Unfortunately, prior MR studies have predominantly focused on traditional cerebrovascular risk factors (e.g., hypertension, dyslipidemia), the causal role of dietary patterns in CSVD pathogenesis remains underexplored. This knowledge gap persists despite growing observational evidence linking diet quality to CSVD progression and the established modifiability of dietary behaviors. Our two‐sample MR analysis evaluated dietary exposures against four main CSVD neuroimaging phenotypes, aiming to disentangle nutrient‐specific causal relationships and inform precision prevention strategies. This study utilized GWAS summary statistics from cohorts such as the UK Biobank to investigate the causal relationship between 32 dietary factors and CSVD‐related phenotypes, including WMH volume, FA, MD, LS, BMB, and PVS.

We hypothesize causal links between specific dietary factors and CSVD neuroimaging biomarkers. Confirmation of these associations would inform modifiable dietary interventions to attenuate subclinical vascular injury, particularly in high‐risk cohorts. By overcoming observational limitations (e.g., residual confounding, exposure misclassification), this MR study uniquely elucidates nutrient‐specific neurovascular mechanisms, thus advancing precision nutrition strategies for targeted CSVD prevention.

## 2 Methods

### 2.1 Study Design

**FIGURE 1:**
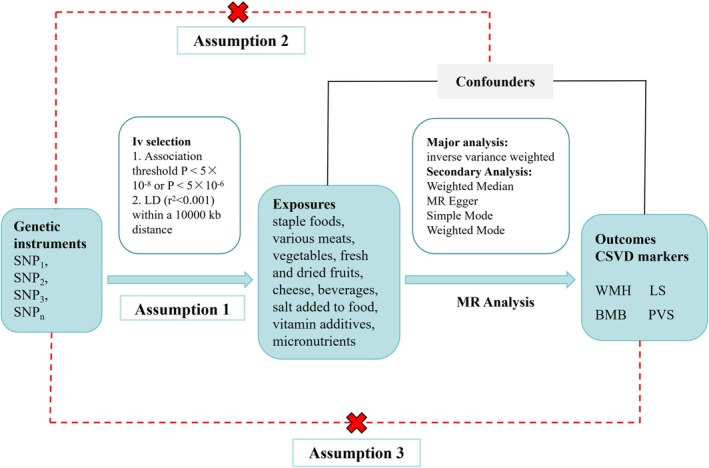
Study design framework and validation of Mendelian Randomization core assumptions. Possible causal relationships between factors that might go against Mendelian randomization assumptions are shown by the dotted arrows.

We used data from the GWAS of diets and CSVD to perform a comprehensive MR analysis using the two sample MR methodology with 32 dietary intakes as exposures and four subtypes of CSVD imaging markers as outcomes to clarify the causal relationship between the two. Following the core assumptions of MR analysis (Lawlor 2016; Evans and Davey 2015), our study requires that IVs be strongly correlated with dietary factors (relevance assumption), independent of potential confounders (independence assumption), and influence the specific neuroimaging markers of CSVD exclusively through those dietary exposures (exclusion restriction assumption). See Figure 1.

Our article was reported according to the STROBE‐MR guideline (Strengthening the Reporting of Observational Studies in Epidemiology‐Mendelian Randomization). All data used in this study are publicly available in their GWAS summary data and therefore do not require ethical approval (Tables 1 and 2).

**TABLE 1:** The positive results of MR analysis for associations between 32 dietary intake and CSVD.

Outcome	Exposure	Method	SNPs	beta	lo_95% ci	up_95% ci	se	*p*‐value	*p*_FDR
WMH volume	PUFA	MR Egger	64	−0.144	−0.248	−0.040	0.053	0.008	0.033
		Weighted median	64	−0.094	−0.175	−0.013	0.041	0.024	0.039
		Inverse variance weighted	64	−0.070	−0.126	−0.015	0.028	0.013	0.033
		Simple mode	64	−0.191	−0.368	−0.015	0.090	0.038	0.047
		Weighted mode	64	−0.110	−0.231	0.011	0.062	0.080	0.080
Any BMB	Iron	MR Egger	10	0.257	−0.027	0.541	0.145	0.114	0.142
		Weighted median	10	0.217	−0.008	0.442	0.115	0.059	0.104
		Inverse variance weighted	10	0.247	0.064	0.430	0.093	0.008	0.041
		Simple mode	10	0.196	−0.150	0.542	0.177	0.296	0.296
		Weighted mode	10	0.226	0.018	0.435	0.106	0.063	0.104
Deep BMB	Iron	MR Egger	10	0.388	−0.090	0.867	0.244	0.151	0.169
		Weighted median	10	0.399	0.025	0.773	0.191	0.037	0.092
		Inverse variance weighted	10	0.414	0.115	0.713	0.153	0.007	0.033
		Simple mode	10	0.467	−0.145	1.079	0.312	0.169	0.169
		Weighted mode	10	0.393	0.024	0.762	0.188	0.067	0.111
Lobar BMB	MUFA	MR Egger	52	−0.493	−0.873	−0.113	0.194	0.014	0.035
		Weighted median	52	−0.278	−0.604	0.048	0.166	0.095	0.135
		Inverse variance weighted	52	−0.298	−0.523	−0.073	0.115	0.009	0.035
		Simple mode	52	−0.312	−0.907	0.283	0.303	0.308	0.308
		Weighted mode	52	−0.263	−0.578	0.052	0.161	0.108	0.135

Abbreviations: any BMB, any brain microbleeds; beta, estimate coefficient; CSVD, cerebral small vessel disease; deep BMB, strictly deep brain microbleeds; lo_95% ci, lower 95% confidence interval; lobar BMB, strictly lobar brain microbleeds; MR, mendelian randomization; MUFA, monounsaturated fatty acid; *P*_FDR, false discovery rate *p* value; PUFA, polyunsaturated fatty acid; se, standard error; SNPs, single nucleotide polymorphisms; up_95% ci, upper 95% confidence interval; WMH volume, white matter hyperintensities volume.

**TABLE 2:** Sensitivity analyses of key Mendelian randomization associations.

Outcome	Exposure	Method	Heterogeneity test		Pleiotropy test		
			Q	*p*_value	Egger_intercept	se	*p*_value
WMH volume	PUFA	MR Egger	71.859	0.184	0.005	0.003	0.106
		Inverse variance weighted	74.978	0.144			
Any BMB	Iron	MR Egger	2.101	0.978	−0.001	0.015	0.928
		Inverse variance weighted	2.109	0.990			
Deep BMB	Iron	MR Egger	7.180	0.517	0.003	0.024	0.895
		Inverse variance weighted	7.198	0.616			
Lobar BMB	MUFA	MR Egger	53.934	0.326	0.013	0.011	0.219
		Inverse variance weighted	55.609	0.305			

Abbreviations: any BMB, any brain microbleeds; deep BMB, strictly deep brain microbleeds; Egger_intercept, MR‐Egger intercept; lobar BMB, strictly lobar brain microbleeds; MUFA, monounsaturated fatty acid; PUFA, polyunsaturated fatty acid; Q, Cochran's Q statistic; se, standard error; WMH volume, white matter hyperintensities volume.

### 2.2 Dietary Exposure Data

Genetic IVs for all dietary exposures were derived from GWAS conducted predominantly in European‐ancestry populations, with summary statistics primarily sourced from the UK Biobank cohort (Rusk 2018). Dietary intake data for most exposures were collected via questionnaires assessing consumption frequency. These included staple foods (e.g., cereals and bread); various meat products (beef, lamb/mutton, pork, oily fish, and non‐oily fish, i.e., lean, low‐fat species such as cod and haddock, poultry, and processed meat); vegetables (raw/salad and cooked); fresh/dried fruits; cheese; beverages (alcohol, coffee, tea, and water); added salt; and vitamin supplements (A, B, C, D, and E). In addition to self‐reported measures, we incorporated circulating biomarkers for essential minerals (iron, copper, zinc, magnesium, calcium, and selenium) (Benyamin et al. 2014) and essential fatty acids (monounsaturated and polyunsaturated fatty acids) (Richardson et al. 2022; Nightingale Health UKBI et al. 2021). These biomarkers were quantified from fasting plasma samples using high‐throughput NMR spectroscopy. Detailed descriptions of all exposures are provided in Table S1.

### 2.3 Outcome Data

Neuroimaging outcome measures were obtained from six recently published large‐scale GWAS investigating MRI‐derived markers of CSVD, which predominantly comprising European populations with rigorously defined diagnostic criteria (Table S1). The outcomes encompassed four primary neuroimaging phenotypes: WMH, LS, BMB, and PVS. Specifically, WMH volume, FA, and MD are indicators related to WMH. PVS is subdivided into white matter PVS (WMH‐PVS; 9317 cases, 29,281 controls), basal ganglia PVS (BG PVS; 8950 cases, 29,953 controls), and hippocampal PVS (HIP PVS; 9163 cases, 29,708 controls) subtypes (Duperron et al. 2023). For BMB, we selected a previously published multicohort GWAS analysis (3556 cases, 22,306 controls), classified as any brain microbleeds (any BMB), strictly deep brain microbleeds (deep BMB), and strictly lobar brain microbleeds (lobar BMB) (Knol et al. 2020). The LS cohort study analyzed 6030 cases and 219,389 controls (Traylor et al. 2021). Although both dietary and outcome data involve samples from European ancestry, the overall difference between calculated exposure levels and outcomes is < 5% (Burgess et al. 2016). GWAS summary statistics for WMH and BMB were retrieved from the Cerebrovascular Disease Knowledge Portal (http://www.cerebrovascularportal.org/), while PVS and LS data were obtained from the GWAS catalog (https://www.ebi.ac.uk/gwas/).

### 2.4 Selection Criteria for Instrumental Variables

Genetic variants associated with dietary intake were sourced from the UK Biobank cohort, which we obtained through the IEU platform (https://gwasmrcieu.ac.uk/datasets). In accordance with three core MR assumptions, we included SNPs at the genome‐wide significant level (*p* < 5 × 10^‒8^) to select valid IVs (Turley et al. 2018). Moreover, we used strict cutoff values (*r* ^2^ < 0.001; region size = 10,000 kb) to remove SNPs that are in linkage disequilibrium (Hemani et al. 2018), aiming to minimize the inclusion of variants in strong linkage disequilibrium that might be related to confounders. Since the application of the strict threshold (*p* < 5 × 10^−8^) yielded fewer than five SNPs for the five vitamin supplements and six micronutrients in this study, a relaxed threshold (*p* < 5 × 10^−6^; *r* ^2^ < 0.001; region size = 10,000 kb) was implemented for these dietary exposures (Turley et al. 2018; Hemani et al. 2018). During the harmonization process, we removed palindromic SNPs and also excluded SNPs significantly correlated with outcomes to satisfy the independence hypothesis (Burgess and Thompson 2011). Finally, to evaluate the IVs intensity, we calculated the F‐statistic (F = beta^2^/se^2^) and retained SNPs with F‐statistic values > 10 to eliminate weak SNP bias (Bowden et al. 2019) (Tables S1, S2). The SNPs that ultimately satisfy all MR hypotheses, including relevance assumption, independence assumption, and exclusion restriction assumption, were selected and used as IVs for further analysis of MR.

### 2.5 Statistical Analysis

This study employed a two‐sample MR framework to systematically evaluate the causal relationship between dietary intake and neuroimaging markers of CSVD. To enhance the robustness of analytical findings, we implemented five complementary MR approaches: inverse variance weighted (IVW), weighted median, MR‐Egger, weighted mode, and simple mode methods. The causal effect estimates derived from MR analyses in this study were expressed as beta coefficients (β) with 95% confidence intervals (CIs). Under the core assumption that IVs must satisfy the “exclusion restriction” criterion (i.e., genetic variants influence outcomes solely through the exposure pathway), the IVW method was designated as the primary analytical approach due to its superior statistical efficiency in the absence of horizontal pleiotropy (Burgess and Thompson 2011). This methodology integrates IVW estimates of SNP‐exposure and SNP‐outcome associations to construct causal exposure‐outcome models (Davey Smith and Hemani 2014). The weighted median estimator provided supplementary robustness by generating reliable causal estimates when up to 50% of instruments were invalid, achieved through ordering SNPs by precision weights and deriving the median of their weighted distribution (Luo et al. 2022). To address potential pleiotropic confounding, MR‐Egger was utilized to detect directional pleiotropy via intercept testing while producing bias‐adjusted effect estimates under heterogeneity (Verbanck et al. 2018). The weighted mode method complemented these analyses by identifying consensus causal effects through SNP clustering patterns (Hartwig et al. 2017).

In this study, the robustness of findings was rigorously validated through sensitivity analyses and false discovery rate (FDR) correction (Sun et al. 2006). A dual‐validation protocol was implemented to ensure result reliability: (1) Statistical significance of IVW estimates was required (threshold: *p* < 0.05); (2) Directional consistency of effect estimates across all supplementary methods with IVW results was strictly enforced. Heterogeneity among individual SNPs was assessed using Cochran's Q test (Bowden et al. 2017), while horizontal pleiotropy was evaluated via MR‐Egger intercept analysis (Burgess and Thompson 2017), with *p*__heterogeneity_/*p*__pleiotropy_ values exceeding 0.05 indicating no significant heterogeneity and pleiotropy, respectively. Leave‐one‐out sensitivity analysis was performed to quantify the influence of individual SNPs by iteratively excluding each variant and recalculating causal estimates. It is worth noting that we also conducted reverse MR analysis to eliminate reverse causality. To ensure the reliability of the research findings, we performed FDR correction for multiple hypothesis testing, which the statistical significance threshold was defined as FDR‐adjusted *p* < 0.05 (Sun et al. 2006). Finally, forest plots, scatter plots, funnel plots, and leave‐one‐out analysis plots were drawn to visualize the results with high confidence. All statistical computations were performed using the TwoSampleMR package (version 0.5.7) in R 4.3.1.

## 3 Results

### 3.1 Dietary Intake and CSVD

**FIGURE 2:**
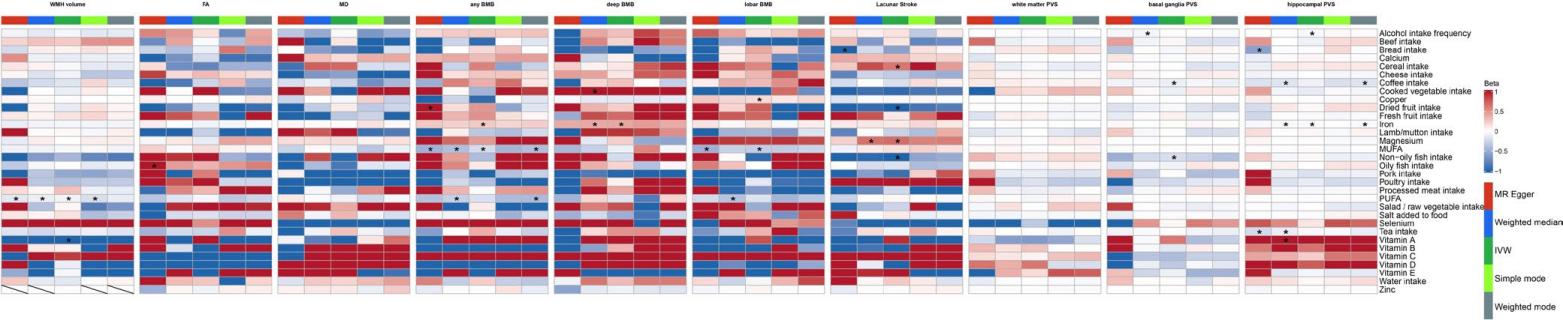
Causal effect estimates of dietary factors on neuroimaging markers of cerebral small vessel disease. Effect sizes (β) from five MR methods are visualized, with blue (protective) to red (detrimental) color scaling. An asterisk (*) denotes nominally significant associations (uncorrected *p* < 0.05). BMB, brain microbleeds; FA, fractional anisotropy; MD, mean diffusivity; MUFA, monounsaturated fatty acid; PVS, enlarged perivascular spaces; PUFA, polyunsaturated fatty acid; WMH, white matter hyperintensities.

The databases and detailed information related to exposures and outcomes used in this study are shown in Table S1. The specific characteristics of diet related IVs are presented in Table S2, which all IVs F‐statistics are greater than 10, avoiding weak instrumental bias. Of the four significant associations (IVW test, *p* < 0.05) identified between 32 dietary factors and CSVD imaging markers, three causal relationships were identified for BMB, and one was observed for WMH volume (Tables 1 and 2; see Figure 2).

### 3.2 Dietary Factors and WMH

**FIGURE 3:**
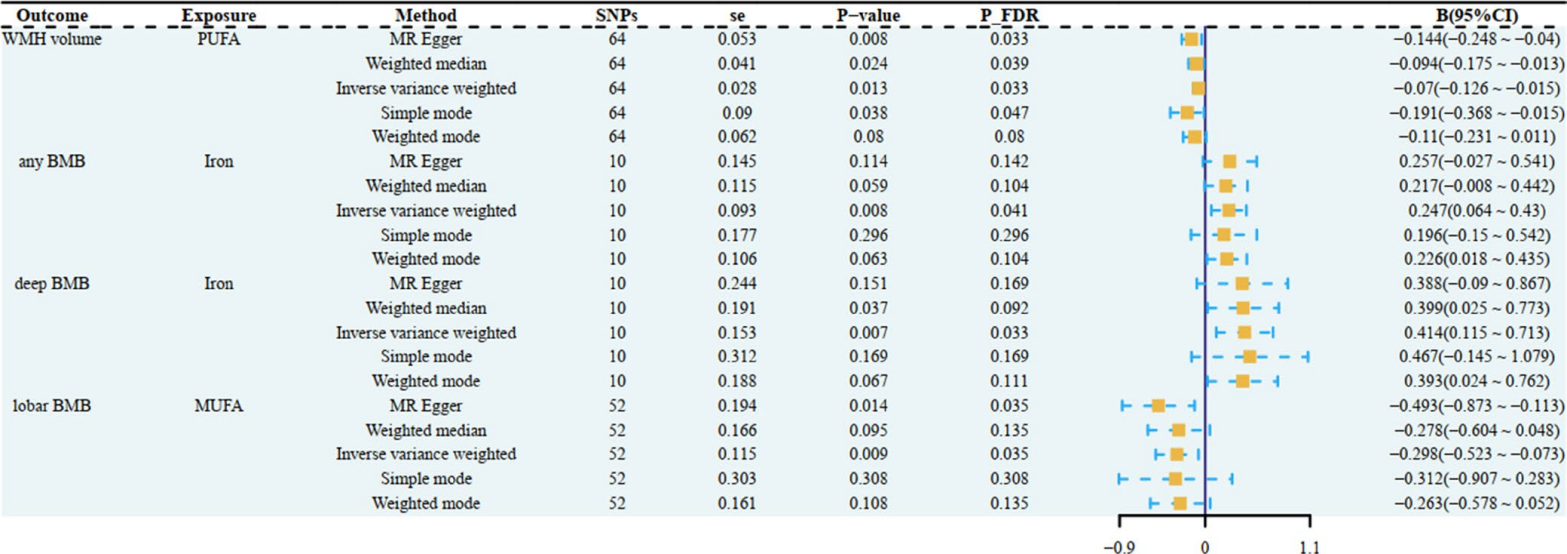
The significant Mendelian randomization (MR) analysis results discovered in the study. WMH, white matter hyperintensities; BMB, brain microbleeds; MUFA, monounsaturated fatty acid; PUFA, polyunsaturated fatty acid; B (95% CI), β coefficient with 95% confidence interval (95% CI); se, standard errors; SNP, single‐nucleotide polymorphism; *p*_FDR, *p* values for false discovery rate correction.

**FIGURE 4:**
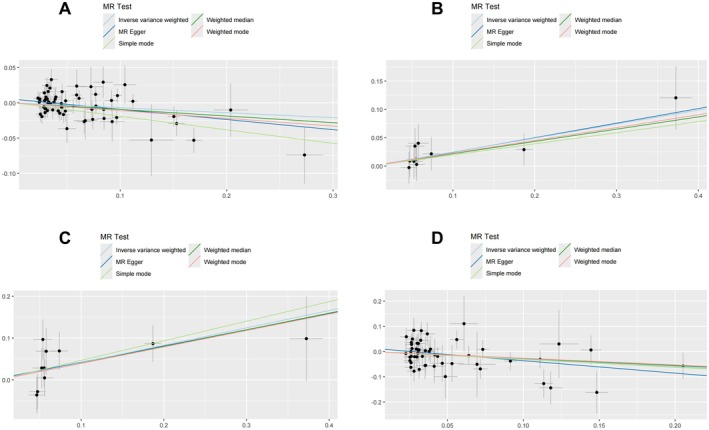
Scatterplots of MR effects between genetically predicted dietary factors and CSVD outcomes. (A) Polyunsaturated fatty acid levels with WMH volume; (B) Iron with any BMB; (C) Iron with deep BMB; (D) Monounsaturated fatty acid levels with lobar BMB.

**FIGURE 5:**
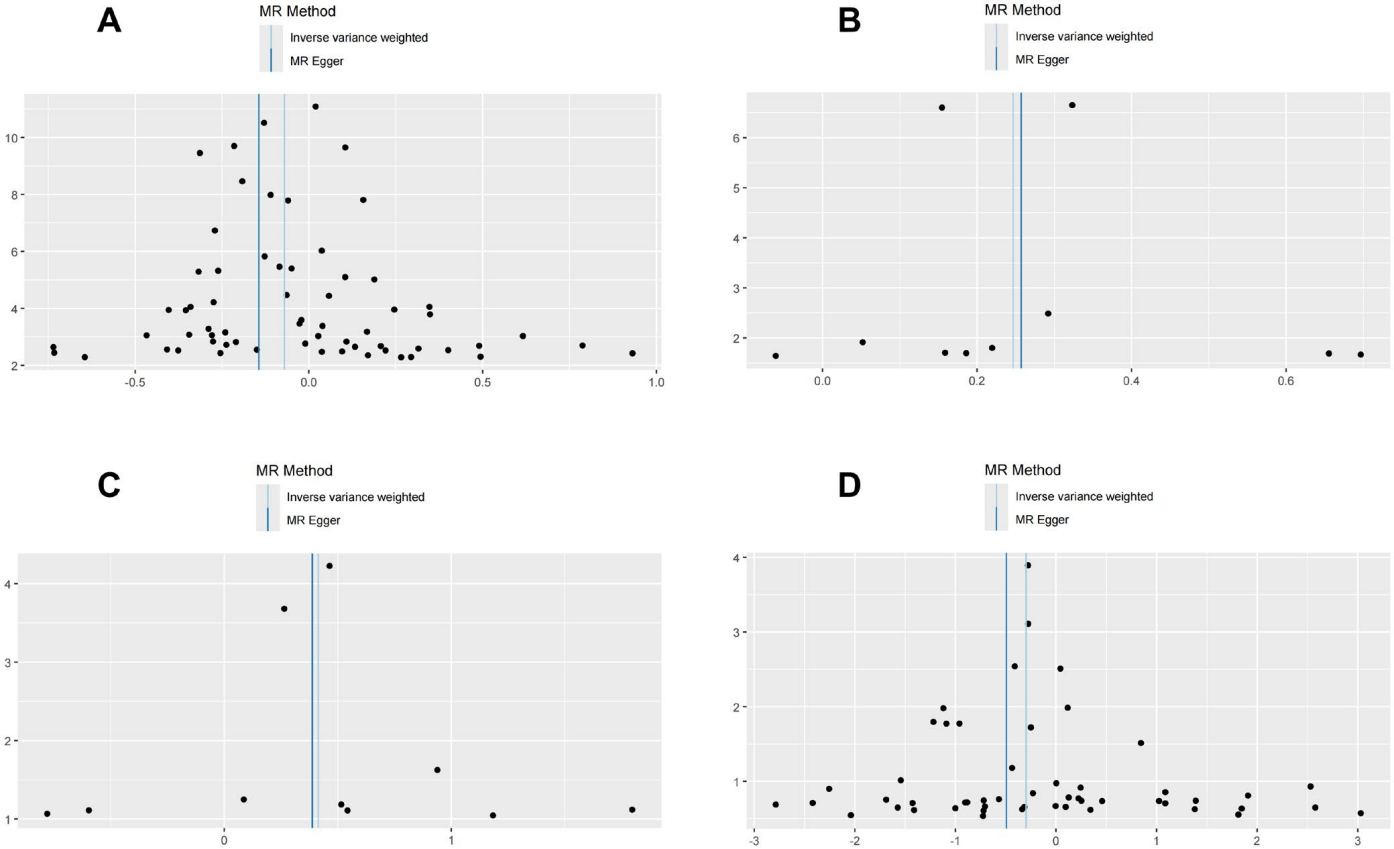
Funnel plots of MR analysis for genetically predicted dietary factors and CSVD outcomes. (A) Polyunsaturated fatty acid levels with WMH volume; (B) Iron with any BMB; (C) Iron with deep BMB; (D) Monounsaturated fatty acid levels with lobar BMB.

**FIGURE 6:**
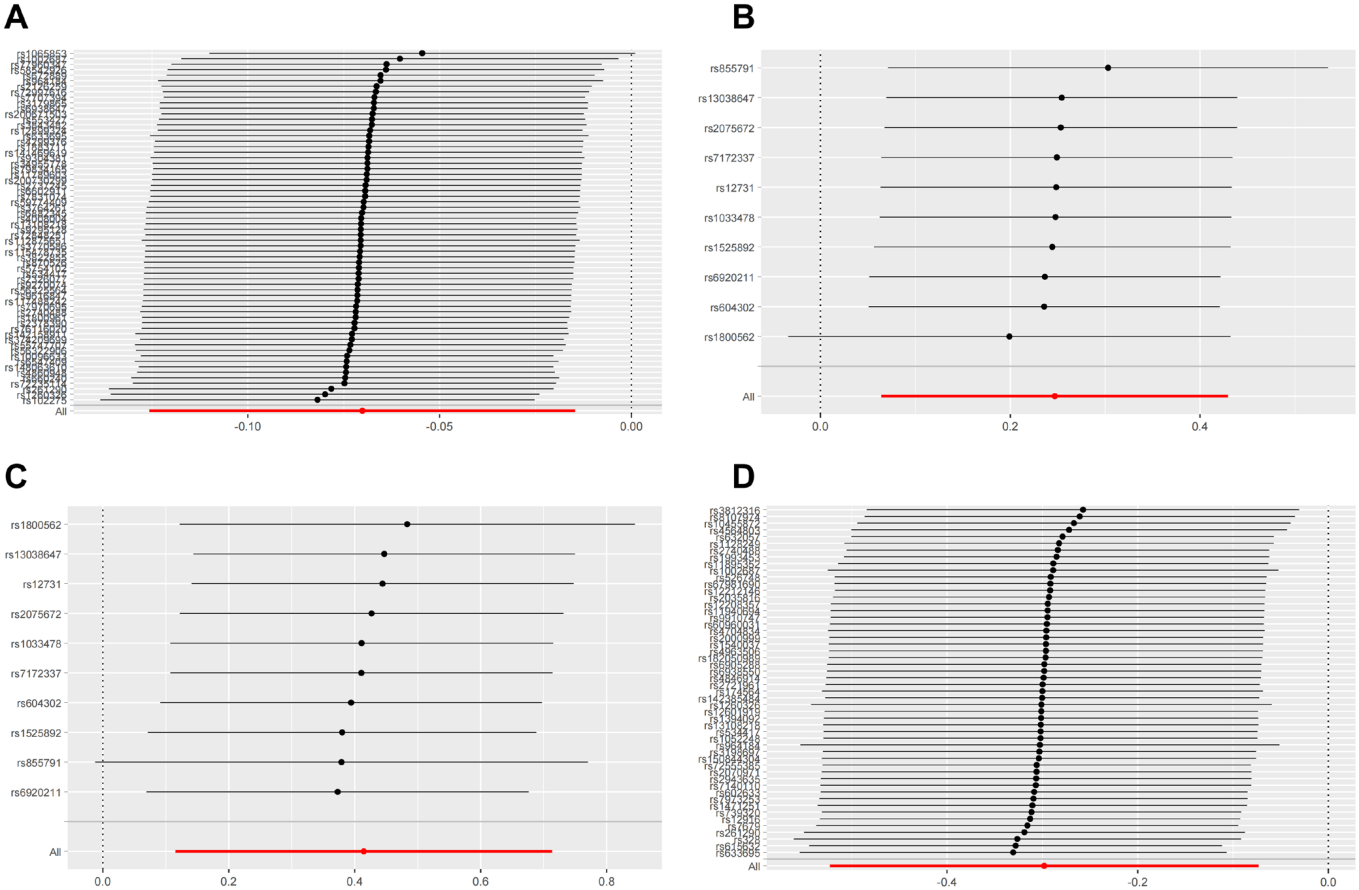
Leave‐one‐out sensitivity analysis of Mendelian Randomization between genetically predicted dietary factors and CSVD outcomes. (A) Polyunsaturated fatty acid levels with WMH volume; (B) Iron with any BMB; (C) Iron with deep BMB; (D) Monounsaturated fatty acid levels with lobar BMB.

In the MR analysis investigating dietary influences on cerebral white matter integrity markers, including WMH volume, FA, and MD, only polyunsaturated fatty acid (PUFA) demonstrated a statistically significant causal relationship with WMH volume. Intriguingly, no causal associations were identified between dietary factors and either FA or MD metrics. The IVW method revealed a significant protective causal effect of genetically predicted PUFA levels on WMH burden (IVW: β = −0.070, 95% CI: −0.126 to −0.015; se = 0.028; *p* = 0.013), which was robust to multiple testing correction using the FDR (FDR‐adjusted *p* = 0.033). Notably, sensitivity analyses employing complementary MR approaches, including MR Egger, Weighted median, Simple mode, and Weighted mode, yielded concordant effect estimates with consistent directionality and statistical significance. The methodological triangulation across these robust approaches strengthened the causal inference, indicating that higher PUFA exposure is reliably associated with reduced WMH burden (Figures 3, 4, 5, 6 and Table S1).

Moreover, Cochran's Q test revealed no significant heterogeneity across the selected instrumental variables for dietary exposures (MR Egger: *Q* = 71.859, *p*__heterogeneity_ = 0.184; IVW: *Q* = 74.978, *p*__heterogeneity_ = 0.144). MR‐Egger analysis further confirmed the absence of horizontal pleiotropy (Egger_intercept = 0.005, se = 0.003, *p*__pleiotropy_ = 0.106). These sensitivity analyses collectively support the robustness of our MR assumptions and reinforce the validity of the observed causal relationships (Tables S3–S5).

### 3.3 Dietary Factors and BMB

MR analyses revealed distinct causal associations between dietary factors and different types of BMB. The results demonstrated a nominally significant negative causal effect of genetically predicted MUFA intake on any BMB risk (IVW: β = −0.205, se = 0.098, 95% CI: −0.397 to −0.013; *p* = 0.036, FDR‐adjusted *p* = 0.045). However, the MR‐Egger intercept test suggested potential horizontal pleiotropy (Egger_intercept = 0.019, *p*__pleiotropy_ = 0.040), and consequently, this association did not meet the sensitivity validation criteria, and we excluded this result. (Figures 2, 3, 4, 5, 6; Tables S12–S14).

Significantly, increased iron intake showed a positive association with any BMB risk (IVW: β = 0.247, se = 0.093, 95% CI: 0.064 to 0.430; *p* = 0.008, FDR‐adjusted *p* = 0.041). For deep BMB specifically, genetically predicted higher iron intake was associated with elevated risk (IVW: β = 0.414, se = 0.153, 95% CI: 0.115 to 0.713; *p* = 0.007, FDR‐adjusted *p* = 0.033). All five MR methods demonstrated complete directional consistency (Directional Consistency Index, DCI = 100%), with no evidence of heterogeneity (for any BMB, MR Egger: Q = 2.101, *p*__heterogeneity_ = 0.978; IVW: Q = 2.109, *p*__heterogeneity_ = 0.990; for deep BMB, MR Egger: Q = 7.180, *p*__heterogeneity_ = 0.517; IVW: Q = 7.198, *p*__heterogeneity_ = 0.616) and horizontal pleiotropy (for any BMB, Egger_intercept = 0.005, se = 0.003, *p*__pleiotropy_ = 0.106; for deep BMB, egger_intercept = 0.003, se = 0.024, *p*__pleiotropy_ = 0.895). In contrast, MUFA levels demonstrated a protective association against lobar BMB (IVW: β = −0.298, se = 0.115, 95% CI: −0.523 to −0.073; *p* = 0.009, FDR‐adjusted *p* = 0.035) and showed no significant heterogeneity (MR Egger: Q = 53.934, *p*__heterogeneity_ = 0.326; IVW: Q = 55.609, *p*__heterogeneity_ = 0.305) or pleiotropy (Egger_intercept = 0.013, se = 0.011, *p*__pleiotropy_ = 0.219). Notably, all five MR methods yielded directionally consistent estimates, and these associations remained statistically significant after FDR correction (Figures 2, 3, 4, 5, 6; Tables S12–S20).

### 3.4 Dietary Factors and PVS

Furthermore, MR analysis identified nominally significant negative causal effects of genetically predicted coffee consumption (IVW: β = −0.088, se = 0.044, 95% CI: −0.173 to −0.002; *p* = 0.045, FDR‐adjusted *p* = 0.225) and non‐oily fish intake (IVW: β = −0.193, se = 0.098, 95% CI: −0.386 to −0.001; *p* = 0.049, FDR‐adjusted *p* = 0.247) on BG PVS. Meanwhile, there is no significant heterogeneity or pleiotropy. However, these associations were disappeared after FDR correction. (Tables S27–S29).

### 3.5 Reverse Mendelian Randomization Analysis

To eliminate the influence of reverse causality on the above results, we conducted a reverse MR analysis using CSVD as the exposure factor and dietary factors as the outcome. The results showed that no reverse causal relationship was found in all positive results, so our MR analysis results are robust (Tables S33–S35). Overall, the positive results retained above were robust. Collectively, these methodological validations indicate that the univariable MR results are robust with minimal bias from pleiotropic effects or outlier variants. The visualization of key results is shown in Figures 2, 3, 4, 5, 6.

Finally, beyond the aforementioned significant associations, our rigorous two sample MR analysis failed to find any significant causal relationships between dietary exposures and other CSVD phenotypes, including FA, MD, WMH‐PVS, HIP‐PVS, and LS (IVW: all *PS* > 0.05) (Tables S3–S32).

## 4 Discussion

To address the limitations of prior studies regarding incomplete dietary factor coverage and phenotypic heterogeneity, we employed a two‐sample MR approach to systematically evaluate causal relationships between 32 common dietary exposures and imaging biomarkers of CSVD. We found that genetically predicted higher PUFA levels were significantly associated with reduced WMH volume, while MUFA had a protective effect on BMB. Besides, increased dietary iron intake demonstrated significant positive causal associations with both any BMB and strictly deep BMB, with these associations remaining statistically significant after FDR correction. Notably, while weak associations were observed between coffee consumption/non‐oily fish intake and BG PVS, these did not survive multiple testing correction. These findings provide novel causal evidence supporting dietary interventions for CSVD prevention and highlight the heterogeneous pathological impacts of distinct nutrients on cerebral small vessels. These findings not only provide new causal evidence for dietary interventions to prevent CSVD but also reveal the heterogeneous characteristics of different nutrients' effects on cerebral small vessel pathology.

As an essential fatty acid, PUFA—categorized into ω‐3 and ω‐6 subtypes—are critical for maintaining cell membrane fluidity and supporting signal transduction (Adili et al. 2018; Wiktorowska‐Owczarek et al. 2015). Observational studies have linked high PUFA intake, particularly from deep‐sea fish, to a reduced risk of cerebrovascular events (Hansen and Burr 1946). These benefits may be partly attributed to the role of PUFA in preserving white matter integrity through anti‐inflammatory and membrane‐stabilizing mechanisms (Kris‐Etherton et al. 2002; Wang and Hu 2017). As integral components of neuronal and glial membranes, PUFA enhance membrane fluidity, thereby facilitating signal transduction, receptor function, and non‐amyloidogenic processing of soluble amyloid precursor protein α (Grimm et al. 2011). Moreover, PUFA exert anti‐inflammatory effects by inhibiting nuclear factor‐ĸ (NF‐κB) pathway, reducing the expression of pro‐inflammatory cytokines such as TNF‐α, IL‐6, and IL‐8 (Hong et al. 2015). Further reinforcing this protective milieu, PUFA suppress MMP‐9 and ADAM17 activity, mitigating BBB disruption—a key process in white matter lesion development (Hong et al. 2015; Chowdhury et al. 2012). At the cellular level, PUFA promote oligodendrocyte maturation and myelination while polarizing microglia toward an anti‐inflammatory M2 phenotype, supporting remyelination and inflammation resolution (Miron et al. 2013; Jiang et al. 2016). Collectively, these mechanisms provide a coherent biological basis for our genetic findings, aligning with observational evidence on the neuroprotective benefits of PUFA‐rich diets such as the Mediterranean diet (Liu et al. 2023; Gardener et al. 2012; Song et al. 2022; Petersson and Philippou 2016). Our results, which establish a causal relationship between PUFA exposure and reduced WMH volume, are consistent with these mechanistic pathways and corroborate previous prospective cohort studies (Chowdhury et al. 2012; Venø et al. 2019). However, a 3‐year randomized controlled trial conducted by Bowman et al. did not find a significant effect of PUFA supplements on WMH progression (Bowman et al. 2019), which may be related to the insufficient duration of intervention to trigger structural imaging changes, suggesting that the neuroprotective effect of dietary nutrition may have a dose‐time accumulation characteristic. It is worth noting that fish are the main source of direct PUFA acquisition, and meta‐analysis has shown that fish intake is associated with a reduced risk of cerebrovascular disease (Yang et al. 2017). Strikingly, this study found a weak association between non‐oily fish consumption and BG PVS, which lost significance after FDR correction. This difference may be due to the pathological mechanism of PVS involving multiple factors such as cerebrospinal fluid dynamics and lymphoid system function, and different studies have heterogeneous operational definitions of “non‐oily fish.” It should be particularly noted that excessive intake of PUFA may produce neurotoxic metabolites through lipid peroxidation reaction (Liu et al. 2010). Therefore, personalized intervention programs should be developed in clinical practice based on individual metabolic characteristics, regional dietary culture, and genetic–nutritional interaction.

MUFA are functional lipids with a single double bond structure, represented by oleic acid, primarily sourced from olive oil and nuts. This study revealed that the genetic prediction of increased MUFA intake had a particularly prominent protective effect on lobar BMB, while no statistical significance was shown in the deep BMB subgroup. This lobar‐specific protective effect may hold particular clinical relevance, as lobar BMB are frequently associated with cerebral amyloid angiopathy, a distinct pathological process within the spectrum of CSVD. MUFA may exert neurovascular protection through both systemic vascular protection and brain microvascular specific regulation. Firstly, MUFA participates in cerebrovascular protection by regulating lipid metabolism by inhibiting the nuclear translocation of sterol regulatory element‐binding protein‐1c (Hunsche et al. 2018). Animal models have also confirmed that MUFA can reduce the area of atherosclerotic plaques in LDL receptor‐deficient mice (Yang et al. 2019). Secondly, MUFA protects the endothelium via dual pathways: directly by activating endothelial nitric oxide synthase to promote NO‐mediated vasodilation, which reduces cerebral microvascular stiffness and mechanical stress; and indirectly by stimulating short‐chain fatty acid‐dependent GLP‐1 secretion, a pathway implicated in anti‐atherosclerosis and endothelial integrity. Together, these effects collectively contribute to the preservation of microvascular integrity and a reduced risk of BMB (Marso et al. 2016; Mason et al. 2018). In addition, MUFA also protects the microvascular structure by regulating oxidative stress and neurovasculitis through multiple targets, such as inhibiting the toll‐like receptor 4/nuclear factor‐ĸB signaling pathway (Hunsche et al. 2018; Finucane et al. 2015). The specific effect of brain microvessels may further explain the targeted protective effect of MUFA on BMB. In terms of blood–brain barrier stability, MUFA maintains the structural integrity of the cerebral vascular basement membrane by inhibiting MMP‐9 activity (Hooper et al. 2020), preventing vascular leakage and microbleeds. Interestingly, MUFA shows no significant protective effect against deep BMB, which may be due to the fact that deep BMB is often caused by hyaline degeneration of deep perforating arteries, a process more dependent on hemodynamic stress rather than the inflammatory‐oxidative injury pathway. Mechanistically, this phenomenon may reflect the heterogeneity in the pathological mechanisms of microvessels across different anatomical sites, or suggest that MUFA primarily exerts its effects through a pan‐vascular protective mechanism, rather than targeting specific etiological pathways.

Besides, we found a positive causal association between iron intake exposure and any BMB and loar BMB risk, which contrasts sharply with previous observational studies that emphasized the vascular protective role of iron (Savarese et al. 2023; Suárez‐Ortegón et al. 2018). Although iron maintains vascular homeostasis by participating in hemoglobin synthesis, mitochondrial energy metabolism, and antioxidant enzyme function (Savarese et al. 2023), this study suggests that iron intake may disrupt the iron metabolic balance in BMB, induce oxidative damage and disruption of the BBB, ultimately leading to BMB. The contradictions between the results of this study and other studies may be due to the biphasic dose effect and mechanism heterogeneity of iron metabolism. Iron is involved in neurotransmitter synthesis, including DA, 5‐HT, as well as myelination, synthesis and oxidative phosphorylation processes (Baker et al. 2010). Within physiological limits, iron maintains homeostasis through the “ferricinile‐iron transport protein axis,” exerting neurovascular protective effects. As age advances, increased iron levels in the brain may be associated with multiple factors, including increased BBB permeability, inflammatory factors, redistribution of iron within the brain, and changes in iron homeostasis (Farrall and Wardlaw 2009). Previous studies have also shown that iron uptake in the brain is bidirectional, but the flow of iron through the BBB is unbalanced, leading to the accumulation of iron in the brain over time (Chen et al. 2014). Iron overload exerts its pathogenic effects primarily through iron‐catalyzed oxidative stress. Excess free iron drives the Fenton and Haber–Weiss reactions, generating highly reactive hydroxyl radicals that overwhelm cellular antioxidant defenses (Galaris et al. 2019; Vinchi 2021). This results in lipid peroxidation, protein dysfunction, and mitochondrial damage, which collectively disrupt microvascular integrity and promote vessel wall rupture, ultimately leading to BMBs (Lee et al. 2012). It is important to note that the brain microvascular system is particularly sensitive to iron imbalance. Iron deposition is present in most BMBs lesions and is significantly associated with the loss of tight junction protein (claudin‐5, occludin) expression in the BBB. The regional susceptibility of deep brain structures to iron‐induced microbleeds may be attributed to several interconnected factors. These include enhanced endothelial iron uptake mediated by transferrin receptor (TfR1) in specific vascular beds (Wiegertjes et al. 2021), the paucity of collateral circulation in deep perforating arteries, and the inherently high metabolic activity of regions like the putamen and globus pallidus. These characteristics collectively increase sensitivity to oxidative stress and compromise vascular resilience, explaining the specific vulnerability to iron‐mediated injury (Li et al. 2022).

Differences in conclusions across studies may further reflect the heterogeneity of baseline iron status and duration of iron exposure in populations. As end‐stage events of CSVD, BMBs may be in the later “window” of iron toxicity accumulation, while most observational studies have not longitudinally tracked the dynamic relationship between iron exposure duration and microvascular lesions. For example, the NHANES study observed a nonlinear protective association of iron clearance against cardiovascular risk (Wiegertjes et al. 2021), but did not stratify the analysis by baseline iron status; the Gutenberg Health study suggested that iron deficiency was associated with all‐cause mortality (Schrage et al. 2020), but its median ferritin levels were significantly lower than the risk threshold in this study. Furthermore, animal experiments also revealed that high iron intake during childhood can accelerate neuronal degeneration by disrupting redox balance (Kaur et al. 2007). These findings have important implications for clinical practice, particularly in individuals at high risk for CSVD, such as those with hypertension or diabetes, where the benefits and risks of dietary iron supplementation need to be carefully evaluated.

The principal strength of this study lies in its MR design, which substantially minimizes reverse causality and confounding biases inherent in observational studies. Unlike previous investigations focusing on single dietary factors, our systematic screening of 32 nutrients/food components enhances statistical power for identifying potential causal associations. Furthermore, stratified analyses across distinct CSVD neuroimaging phenotypes provide novel insights into the pathotype‐specific effects of dietary exposures. The adoption of objective neuroimaging endpoints, superior to symptom‐based assessments, effectively reduces recall bias and diagnostic heterogeneity. However, several limitations warrant cautious interpretation. First, the exclusive reliance on European‐ancestry genomic data limits generalizability to populations with divergent dietary patterns and genetic architectures, particularly given known ethnic variations in nutrient metabolism (e.g., lactase persistence alleles). Second, while rigorous instrument selection and sensitivity analyses were implemented, residual horizontal pleiotropy cannot be entirely excluded. Notably, despite the robustness of the iron intake analysis—supported by a high F‐statistic and consistent effect estimates across MR methods—its statistical power was potentially limited by the number of available genetic instruments (10 SNPs). Confirmation of these findings with greater precision thus awaits future GWAS with larger sample sizes. Third, although MR captures lifelong exposure effects, it cannot delineate critical time windows for neurotoxicant accumulation which a key concern for iron's cumulative cerebrovascular toxicity observed in preclinical models. Based on these findings, we propose future research directions, conduct dose–response studies to establish optimal intake thresholds for PUFA/MUFA/iron in CSVD prevention, leveraging nutrient biomarker‐guided interventions. Investigate gene‐diet interactions (e.g., HFE C282Y mutations modulating iron's cerebrovascular effects) to enable risk‐stratified interventions. Employ advanced neuroimaging techniques (e.g., dynamic contrast‐enhanced MRI) to quantify real‐time BBB modulation by dietary interventions. Integrate dietary patterns into multimodal machine learning models (incorporating genomics, imaging, and metabolomics) to refine CSVD early detection algorithms.

## 5 Conclusion

This two‐sample MR study provides novel genetic evidence supporting causal relationships between dietary patterns and CSVD manifestations. Three key findings emerge with clinical relevance: higher PUFAs intake demonstrates protective associations against WMH volume, potentially mediated through anti‐inflammatory pathways and endothelial stabilization. Similarly, a negative causal effect of MUFA on lobar BMB was observed, supporting their postulated role in maintaining BBB integrity. In contrast to observational studies emphasizing the vascular protective role of iron, our MR analysis indicated that genetically predicted iron levels were positively associated with the risk of both any and deep BMB, suggesting a potential adverse role. The diverging effects between unsaturated fats and iron supplementation underscore the necessity of nutrient‐specific dietary interventions for CSVD prevention. Mediterranean‐style diets rich in unsaturated fats but moderate in iron‐fortified foods may optimize neurovascular benefits. These findings should be interpreted considering inherent limitations: population stratification (European‐biased genetic instruments), time‐varying exposure effects unaddressed by MR, and unresolved mechanisms linking specific nutrients to CSVD pathobiology. Future randomized trials targeting PUFA/MUFA supplementation and iron restriction in high‐risk populations, coupled with multimodal neuroimaging biomarkers, are warranted to validate these causal associations.

## Author Contributions

Y.Z., Y.Z., H.L., Z.Z., S.X., S.N., Y.Z., and Z.Z. participated in the concept, design, and writing articles. H.L. and Y.Z. obtained and validated the data used in the study. S.X. analyzed the data. S.N. participated in the conceptualization, review, and editing of this article. All authors have contributed to the article and approved the submitted version.

## Funding

The authors have nothing to report.

## Consent

The authors have nothing to report.

## Conflicts of Interest

The authors declare no conflicts of interest.

## Supporting information


**Table S1:** fsn371374‐sup‐0001‐Tables.xls.

## Data Availability

All data used in this Mendelian randomization study are publicly available summary statistics from genome‐wide association studies. The specific data sources and accession identifiers are listed in Table S1.
